# Online Role-Play in Palliative Care Education for Early-Career Nurses in Japan: A Pre–Post Evaluation of Perceived Impact and Feasibility

**DOI:** 10.1177/10966218251371124

**Published:** 2025-09-18

**Authors:** Anri Inumaru, Tomoko Tamaki, Mayumi Tsujikawa, Jun Kako

**Affiliations:** ^1^ Department of Nursing, Graduate School of Medicine, Mie University, Tsu, Japan.; ^2^ Department of Fundamental Nursing, Shiga University of Medical Science, Otsu, Japan.; ^3^ Faculty of Nursing, Suzuka University of Medical Science, Suzuka, Japan.

**Keywords:** distance, education, Japan, nurses, palliative care, role-playing

## Abstract

**Background:**

As the global incidence of cancer rises, high-quality palliative care is increasingly needed. Early-career nurses often find symptom management and communication challenging. Although role-playing is a promising educational approach, its effectiveness in fully online formats remains underexplored.

**Objective:**

This study aimed to examine the feasibility and preliminary effectiveness of a fully online, role-play-based palliative care education program and to explore participants’ experiences with this program.

**Methods:**

This prospective observational study included 31 nurses with 2–4 years of practice. Participants completed assessments before, right after, and one month after the intervention. These assessments included the Palliative Care Difficulties Scale, Nurses’ Difficulty with Cancer Care, Palliative Care Self-reported Practices Scale, confidence, practice level (with reference to the Objective Structured Clinical Examination), and satisfaction. Furthermore, qualitative feedback was collected one month after the program.

**Results:**

Perceived difficulty in alleviating symptoms and communicating with the patient and family significantly decreased one month after the intervention. Confidence and selected practical communication behaviors (e.g., “Support” and “Explore”) also improved. Participants reported high satisfaction, and over half of them described applying skills in practice.

**Conclusion:**

Online role-play-based training is a feasible and acceptable strategy to help early-career nurses develop palliative care competencies. Further research using randomized controlled trials is warranted to establish its effectiveness.

## Introduction

The incidence of cancer continues to increase globally,^1^ underscoring the growing urgency for high-quality palliative care.^2^ Basic palliative care is a fundamental responsibility of all health care professionals.^3^ However, less experienced nurses often struggle with symptom management and communication, which are key aspects of palliative care.^4–6^ These difficulties reduce clinical performance and contribute to burnout.^6,7^ Therefore, palliative care skill-building strategies are needed for early-career nurses.

The End-of-Life Nursing Education Consortium (ELNEC) program is a widely adopted framework for palliative care education.^8^ With increased Internet access, online education has grown in popularity,^9^ offering advantages including time efficiency and high learner satisfaction.^10^ Although the ELNEC program is available to nursing students online,^11^ it provides only passage lecture-based education and lacks interactive features that engage learners. In Japan, most nursing education in end-of-life care is provided through the End-of-Life Nursing Education Consortium-Japan (ELNEC-J). It is included in the continuing education provided for clinical nurses. General nurses deliver primary palliative care in settings such as acute wards, long-term care facilities, and patients’ homes, while specialized teams provide advanced palliative care in cancer centers and palliative care units.

Role-play is an educational strategy that can effectively enhance communication, clinical skills, and self-confidence in palliative care nurses.^12–14^ However, these programs are typically delivered face-to-face, and the feasibility of online role-play programs has not been sufficiently evaluated. Face-to-face programs may be difficult for working nurses to attend. Online education improves accessibility by allowing participants to study anywhere.^15^ Given the ongoing shift toward digital education, evaluating the potential of fully online, interactive palliative care education is timely and important.^16^ Therefore, this study addresses the major gaps in the literature on the feasibility and perceived changes of online role-play-based palliative care education for nurses. This study aims to investigate changes in the difficulties experienced by early-career nurses in symptom management and communication, and their confidence and skill application. It also aims to explore the feasibility of an online, role-play-based palliative care education program.

## Materials and Methods

### Design

We employed a pre–post, single-arm design. Nurses were recruited from multiple facilities in the Tokai region of Japan. Eligibility criteria were nurses with two to four years of clinical experience, those who signed the research participation consent form, and those able to participate online. Exclusion criteria were those whose native language was not Japanese. We sent requests to each facility requesting the recruitment of research participants. Interested nurses were then sent a description of the study and a consent form. They were asked to return the completed consent form if they wished to participate. The study was approved by our ethics review committee (no. U2022-014).

### Operational definition of terms

The study’s palliative care education, which is based on previous research,^4,5,17^ included content related to total pain management and communication.

### Setting and participants

This study targeted nurses with two to four years of clinical experience working in hospitals across the Tokai region, including designated cancer treatment base hospitals, quasi-base hospitals, and affiliated institutions with a certified nurse specialist (CNS) in cancer nursing. After reviewing the study details, participants provided informed consent and obtained access to an online environment to attend the program.

### Development

An online palliative care education program incorporating role-play was designed based on previous research^17^ to help develop competencies in pain management and communication in early-career nurses. The ELNEC-J framework was employed as a reference. The educational program development team consisted of three nursing researchers with at least five years of experience each in palliative care education, one nurse specialist in cancer nursing, and one certified palliative care nurse. The team ensured that the program was of an appropriate level of difficulty for nurses in their second to fourth years and that the cases reflected likely real-world scenarios. Each session was 90 minutes long. This combined orientation, role-play, and mini-lecture was based on the palliative care training program of the Japanese Society of Psycho-Oncology.^18^ A pilot test was conducted before the main study.

Participants received advance handouts that outline the case and key concepts. The learning objectives were as follows: (1) to recognize patients’ total suffering and (2) to communicate appropriately according to the patients’ condition. The main case scenario involved a patient with advanced cervical esophageal cancer experiencing lumbar metastasis-related pain. We informed participants in advance how to conduct an online connection test.

### Measurements

1. Palliative care difficulties scale (PCDS)
  This six-item scale assesses perceived difficulty in “alleviating symptoms” and “communication with patients and families” using a five-point Likert scale (1 = strongly disagree to 5 = strongly agree), demonstrating strong reliability and validity. Higher scores (range: 3–15 per subscale) indicate greater difficulty. Cronbach’s α was 0.87 for symptom alleviation and 0.91 for communication. This scale was chosen because these domains are particularly difficult for early-career nurses.^5^
2. Nurses’ difficulty with cancer care (NDCC)
  The scale uses a six-point Likert scale (1 = strongly disagree to 6 = strongly agree) to assess the degree of difficulty in 13 items related to “communication,” showing high validity and consistency. Higher scores (13–78 for each subscale) indicate a higher degree of difficulty. Cronbach’s α for the communication subscale was 0.69. This tool was used to assess communication difficulties in cancer care, as communication is a key aspect of palliative nursing. The measure complements the PCDS by providing a more detailed evaluation of communication challenges.^19^
3. Palliative care self-reported practices scale (PCPS)
  This three-item scale assesses the degree of implementation in “pain” and “communication” using a five-point Likert scale (1 = not done, 5 = always done), with high reliability and validity. Higher scores (3–15 for each subscale) indicate a higher degree of implementation. Cronbach’s α was 0.91 for the pain subscale and 0.80 for communication. This scale was selected to measure pain management and communication behaviors in practice and to evaluate changes in their implementation frequency after the training.^5^
4. Level of practice (in evaluating researchers)
  Developed with reference to the Objective Structured Clinical Examination (OSCE) method and prior research, this evaluation tool consists of 14 binary items assessing three domains: physical assessment (5 items), communication (4 items), and communication with NURSE (5 items). Higher total scores indicate greater observed competence.
  Before and after the intervention, the participants read the case and the assignment and provided care to the simulated patient online. Requested from the Mie Simulated Patient Association, the simulated patients were played by individuals who are regularly involved in medical education and have experience with OSCE. These simulated patients were given materials in advance to prepare for their roles, and a two-hour meeting was held. In the Physical Assessment domain, nurses were instructed to provide physical assessment and palliative care for physical pain for four minutes to a patient who previously underwent lung cancer surgery, had a metastasis in the thigh, and was admitted for pain management. The Communication and Communication Using NURSE domains required the participants to engage in four minutes of appropriate communication with a patient who had been living with a stoma for five years following rectal cancer surgery and who had been informed by their physician the previous day that the chemotherapy was not effective. NURSE is a communication technique requiring nurses to have empathy while responding to patients. It has five elements: Name, Understand, Respect, Support, and Explore. Then, two or more palliative care educators, including an oncology CNS, used the recorded care scenes as study data and evaluated them using a binary scale (1 = achieved, 0 = not achieved).
5. Confidence
  Based on previous studies,^20^ this scale uses a six-point Likert scale (1 = not at all disagree 6 = strongly agree) to assess the degree of confidence in “physical assessment” and “perception of holistic distress.” The higher the scores (1–6 on each subscale), the greater the confidence.
6. Satisfaction
  Based on previous studies,^21^ this scale uses a six-point Likert scale (1 = strongly disagree to 6 = strongly agree) to assess the degree of satisfaction. This tool evaluates aspects such as the clarity of objectives, training relevance, environment, and peer reflection.
7. Qualitative data
  The content of the comments section, which asked about “things that were felt or utilized in the past month,” was analyzed using Berelson’s content analysis as a reference.^22^ Such content was collected one month later. A single sentence was used as the unit of record and a paragraph as the unit of context. Each sentence was then compared, classified, and named according to the similarity of its semantic content. To increase the reliability of the results, two researchers examined the entire process. Qualitative data were collected to explore the individual perspectives of participants in more depth, facilitating a more comprehensive assessment of the impact of the intervention.

## Procedure

Participants were recruited through their affiliated facilities. After understanding the study information sheets, interested participants provided written informed consent. Immediately before the training, eligible participants were assessed using PCDS, NDCC, PCPS, level of practice, and confidence to collect baseline data. Immediately after the training, all such assessments were conducted again, and the participants were asked to enter their level of satisfaction. The same assessments, excluding level of practice (in evaluating researchers), were reconducted one month later, and the participants were asked to enter their impressions of how they had actually used them.

The role-play method was based on the Palliative Care Training Program of the Japanese Society of Psycho-Oncology. The exercise, which lasted for 90 minutes, was conducted in the following order: (1) orientation, (2) deciding on a scenario and preparing for the role, (3) role-play, (4) feedback, (5) repeating (2) to (4) two to three times, and (6) sharing with the whole group. In each group, three to four participants took turns in playing the roles of nurse, patient, and observer. During the orientation, they were given their goals; one goal was to focus on their own awareness and communication. During the role-playing, the participants acting as the patient were given time to prepare for their role and think about the patient. In the first and second sessions, the participants were asked to act as a patient with a strong sense of pain (physical pain, mental pain, social pain, and spiritual pain). Those acting as nurses communicated with the patient with a specific objective in mind (e.g., what kind of communication skills to use). The observers observed the role-play and provided feedback, focusing on the good points of the nurse role. One facilitator participated in each group. Qualified facilitators were those who had been involved in cancer nursing education for more than five years. The role-play was conducted using an online tool.

### Sample size

Given that the effect size of previous research was unclear, we used Cohen’s criteria. With a two-tailed test, *d* = 0.4 (medium to large effect size), a probability of α error of 0.05, and a probability of β error of 0.2, the required sample size was 27. Assuming a 10% dropout rate, the researchers set the target sample size at 30.

### Statistical analysis

The primary outcome was the change in PCDS from the baseline (pre-intervention) to one month after the intervention. The secondary outcomes included changes in NDCC, PCPS, and confidence from the baseline to one month after the intervention. Within the same period, they also included changes in the level of practice in evaluating researchers; satisfaction after the intervention; and impressions one month after the intervention. Patients with missing data of 20% or more were excluded. The number of people and their proportions were shown for basic characteristics. Descriptive statistics were calculated for all items. All statistical analyses were performed with Easy R (EZR) (Jichi Medical University, Tochigi, Japan), which is a graphical user interface for R (The R Foundation for Statistical Computing, Vienna, Austria).^23^ The Friedman test was employed for the three time points (before, after, and one month after the interventions). The two time points after the intervention were evaluated using the Wilcoxon signed-rank text. For assessing the level of practice in evaluating researchers, McNemar’s test was employed. The significance level was set at less than 0.05. The effect size was calculated using Cohen’s *d* with G*power 3.1.9.7.^24^ The results of the satisfaction survey are shown as means and standard deviations (SD).

## Results

### Participant flow and baseline characteristics

**FIG. 1.**
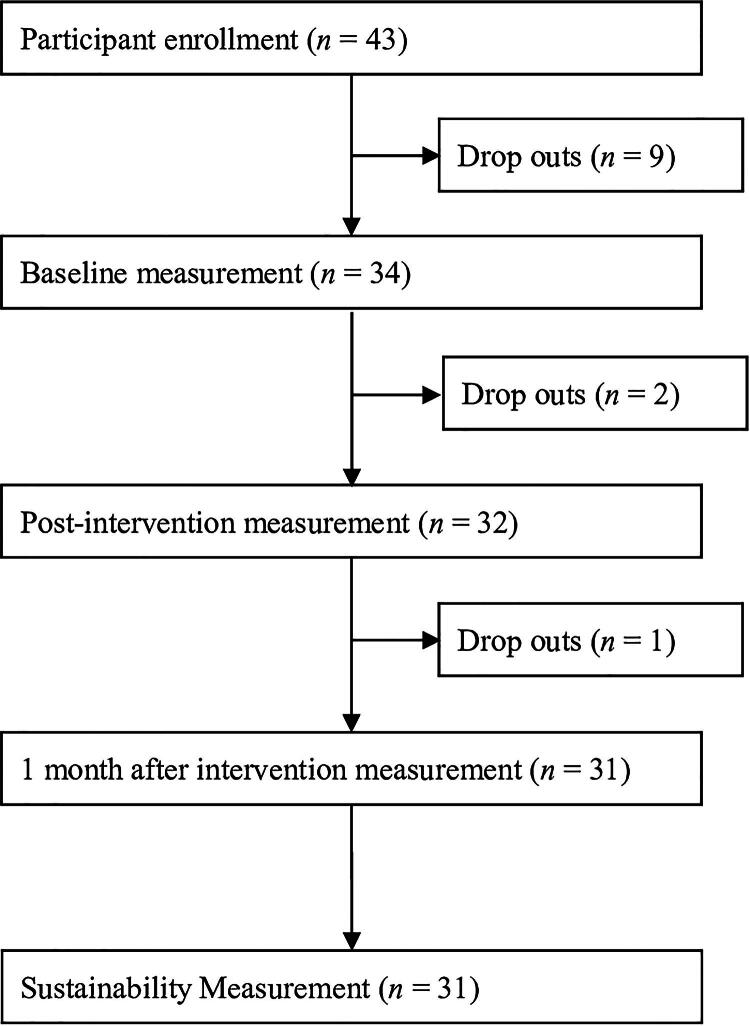
Flowchart of the study.

Data were collected between July 2022 and August 2023. Of the 43 people interested in participating, 31 were included (Fig. 1). Participants dropped out from the survey before the baseline measurement, mostly because they were not contacted, forgot their appointment date, had no data as a result of poor Internet access, were absent resulting from equipment failure, and had no data excuse for being late on the day (*n* = 13, 2, 2, and 1, respectively). The reason for dropping out after the intervention was that two participants left early; thus, no data were available. The reason for dropping out one month later was that one participant could not be contacted.

Four facilities participated. More than half of the participants had graduated from vocational nursing schools, and none had previously received formal palliative care education. Table 1 lists the participant characteristics and baseline.

**Table 1.** Characteristics of the Participants

	*N* = 31 Mean (SD)
Age (years)	23.8 (1.0)

	*n* (%)
Educational status	
Associate’s degree (3 years)	17 (54.8)
College degree	14 (45.2)
Experience in palliative care training	
None	31 (100)
Clinical experience (years)	
2	13 (41.9)
3	10 (32.3)
4	8 (25.8)
Number of caring for patients with cancer (per year)	
0	6 (19.4)
1–9	7 (22.6)
10–49	12 (38.7)
50–99	4 (12.9)
>100	2 (6.5)

SD, standard deviation.

### Changes in difficulty, practice, and confidence

Table 2 presents the mean and standard deviation values of PCDS, NDCC, PCPS, and confidence scores at baseline (T0), immediately after the intervention (T1), and one month later (T2). For the primary outcome (PCDS), the perceived difficulty in alleviating symptoms (*p* = 0.002, *d* = 0.694) and communication with patient and family (*p* = 0.048, *d* = 0.492) significantly improved at T2. Conversely, NDCC and PCPS showed no significant differences between T1 and T2. For the two learning objectives, participants’ confidence in noticing total pain (*p* = 0.003, *d* = 0.877) and in communication with patients (*p* < 0.001, *d* = 1.094) improved significantly.

**Table 2.** Summary of Endpoints Before, After, and One Month After the Educational Program

Measure	T0	T1	T2	*p* ^a^	*p*^b^/Cohen’s *d*		
	M/(SD)	M/(SD)	M/(SD)		T0 vs. T1	T0 vs. T2	T1 vs. T2
Primary endpoint							
Palliative care difficulties scale (PCDS)							
Alleviating symptoms	3.58 (0.79)	3.28 (0.78)	3.05 (0.73)	0.000^**^	0.146/0.385	0.002^**^/0.694	0.126/0.300
Communication with the patient and family	3.72 (1.06)	3.55 (0.86)	3.26 (0.73)	0.097	0.567/0.176	0.048^*^/0.492	0.220/0.360
Secondary endpoint							
Nurses’ difficulty with cancer care (NDCC)							
Communication	56.35 (13.43)	55.61 (9.79)	53.61 (11.49)	0.236	>0.999/0.062	0.813/0.218	0.702/0.186
Confidence							
Noticing total pain	2.32 (0.98)	3.19 (0.79)	3.13 (0.85)	0.000^**^	0.002^**^/0.967	0.003^**^/0.877	>0.999/0.079
Communicating with patients	1.94 (0.96)	3.00 (0.82)	2.94 (0.85)	0.000^**^	0.000^**^/1.184	0.000^**^/1.094	>0.999/0.077
Palliative care self-reported practices scale (PCPS)							
Pain	3.55 (0.96)	—	3.87 (0.76)			0.159/0.367	
Communication	3.46 (0.91)	—	3.59 (0.71)			0.842/0.156	

*p*-Value: ^a^Friedman test, ^b^Wilcoxon signed-rank test (Bonferroni correction). ^*^p < 0.05, ^**^p < 0.01 (statistical significance).

Effect size (Cohen’s *d*): *d* > 0.5 (medium), *d* > 0.8 (large).

T0: before, T1: after, T2: one month after.

### Level of practice in evaluating researchers

Table 3 shows the percentage of practice in evaluating researchers at T0 and T1. In the Pain assessment, the “Dealing with Misunderstandings about Medical Narcotics” item exhibited a significant change (*p* = 4). In the NURSE communication, a significant difference was noted in the “Supporting” (*p* < 0.001) and “Exploring” (*p* = 0.021) items.

**Table 3.** Outcome: Level of Practice in Evaluating Researchers

Measure			T1		*p* ^a^
			Can (%)	Cannot (%)	
Pain assessment					
Checking patient’s pain intensity using pain scales such as Numerical Rating Scale (NRS), Face Scale, and Visual Analogue Scale (VAS)	T0	Can	3 (9.7)	6 (19.4)	0.289
		Cannot	2 (6.5)	20 (64.5)	
Checking for daily activities interfered with pain		Can	10 (32.3)	9 (29.0)	0.423
		Cannot	5 (16.1)	7 (22.6)	
Identifying drowsiness, constipation, and nausea as side effects of medical narcotics		Can	22 (71.0)	4 (12.9)	0.683
		Cannot	2 (6.5)	3 (9.7)	
Checking the situation of when the pain appeared		Can	22 (71.0)	5 (16.1)	0.724
		Cannot	3 (9.7)	1 (3.2)	
If the patient has misconceptions about narcotics (addiction, gradual loss of effect, strong side effects, etc.) when starting narcotics, correct knowledge is explained to the patient		Can	22 (71.0)	6 (19.4)	0.041^*^
		Cannot	0 (0)	3 (9.7)	
Communication					
Setting up a quiet and private place	T0	Can	22 (71.0)	4 (12.9)	0.371
		Cannot	1 (3.2)	4 (12.9)	
Using open queries		Can	1 (3.2)	6 (19.4)	0.752
		Cannot	4 (12.9)	20 (64.5)	
Repeating what the patient says in his or her own words		Can	6 (19.4)	7 (22.6)	0.773
		Cannot	5 (16.1)	13 (41.9)	
Enduring short silences (5–10 seconds)		Can	15 (48.4)	5 (16.1)	1
		Cannot	6 (19.4)	5 (16.1)	
NURSE communication					
Naming	T0	Can	12 (38.7)	6 (19.4)	0.343
		Cannot	4 (12.9)	9 (29.0)	
Understanding		Can	17 (54.8)	6 (19.4)	0.752
		Cannot	2 (6.5)	6 (19.4)	
Recognizing		Can	9 (29.0)	14 (45.2)	0.289
		Cannot	0 (0)	8 (25.8)	
Supporting		Can	0 (0)	9 (29.0)	0.000**
		Cannot	1 (3.2)	22 (71.0)	
Exploring		Can	12 (38.7)	6 (19.4)	0.021*
		Cannot	4 (12.9)	9 (29.0)	

*p*-Value: ^a^McNemar test was conducted. ^*^p < 0.05, ^**^p < 0.01 (statistical significance).

### Satisfaction with the educational program

Table 4 shows the satisfaction levels. The mean score for all 10 items was 4.9 points. Some of the scores obtained were as follows: “The level of difficulty of the training was appropriate,” 4.74 (SD = 0.77); “The time was neither too long nor too short,” 4.65 (SD = 0.84); “There were no problems with the implementation environment,” 4.68 (SD = 0.91); and “I was able to participate with a moderate sense of tension,” 4.84 (SD = 0.69). These results indicate that the participants were highly satisfied with the training.

**Table 4.** Satisfaction

	Mean	SD
Today’s training was satisfactory	4.90	0.75
The training content was appropriate for my needs	4.77	0.99
The learning objectives were clear	4.94	0.73
The level of difficulty of the training was appropriate	4.74	0.77
No problems with the environment emerged	4.68	0.91
The duration of the training was reasonable, neither too long nor too short	4.65	0.84
The instructor’s involvement was supportive of learning	4.97	0.71
The level of tension was appropriate	4.84	0.69
The handouts provided in advance aided in learning	4.74	0.89
I was able to discuss with my peers and analyze what was good and what was lacking	4.90	0.70

### Qualitative feedback one month after training

Table 5 shows the results of the content analysis of the impressions. Seventy codes were extracted. The most common codes were as follows: “Putting into practice what was learned in the training” (58.6%, 41 codes), “Reviewing the training” (15.7%, 11 codes), “Learning” (11.4%, 8 codes), “Not yet put into practice what was learned in the training” (8.6%, 6 codes), and “Future prospects” (5.7%, 4 codes).

**Table 5.** Impressions One Month After the Intervention

Category	Subcategory	Number of codes		Participant’s comments (participant no.)
Putting into practice what was learned in the training	Practicing communication with awareness of its importance	41	17	I could listen to the patient’s thoughts and feelings and have an empathetic attitude. (24)
	Putting into practice pain management		7	I began to write down in detail the pain intensity, the situation, and the evaluation after the patient received painkillers. (23)
	Encountering difficulties in practice		5	Immediately after the diagnosis, the patient was quite shocked, so interaction at that time was difficult. (2)
	Practicing communication with family members		5	I could think of a plan with the family to play the patient’s favorite music and implement it. (37)
	Focusing on holistic pain		3	After this training, I could interact with patients who were worried about their future treatment and acknowledge their pain by imagining their holistic suffering. (36)
	Practicing sharing between health care professionals		3	I began to actively share the information I had gained at conferences and listen to opinions about intervention methods from different perspectives. (31)
	Practicing with an awareness of the overall training		1	During the first or second week after the training, I was conscious of what I had learned. (12)
Reviewing the training	Reviewing communication	11	7	The training allowed me to reflect on my own communication methods. (22)
	Reviewing the training holistically		2	I’m glad I was able to participate in the training. (19)
	Reviewing holistic pain		1	Through this training, I could reflect on what holistic suffering is, and it was an opportunity to reconsider it. (24)
	Reviewing pain management		1	Until now, I think I’ve lacked confidence in my own responses when patients express their suffering, and I think I’ve shown my confusion. (36)
Learning	Learning communication	8	6	By taking this training, I could think about what kind of questions and words I would want to hear if I were the patient. (31)
	Learning pain management		2	I felt that writing down the degree of pain in a way that was easy to understand and specific is necessary because the degree differs from person to person. (3)
Not yet applying what was learned in the training	Lacking opportunity to put into practice what was learned in the training	6	5	I learned communication methods, but I wasn’t able to create a situation where I could use them. (5)
	Feeling too busy to put into practice what was learned in the training		1	After three weeks, I was too busy to be aware of some things. (12)
Future prospects	Future prospects	4	4	I strongly felt that I wanted to become a nurse who could draw out the thoughts of the patient, and I want to reflect on what I learned in the training again. (10)

## Discussion

This study demonstrated that a fully online, role-play-based palliative care education program significantly reduced early-career nurses’ perceived difficulty in alleviating symptoms and communication with the patient and family while also improving their confidence in clinical practice. High levels of participant satisfaction and qualitative feedback further support the program’s effectiveness.

Importantly, the participants’ PCDS scores significantly improved one month after the intervention, particularly in the alleviating symptoms domain, which is challenging for novice nurses according to previous studies.^5^ Although role-playing is effective in developing communication skills,^25,26^ our findings suggest that this method also enhances perceived competence in alleviating symptoms. This is especially relevant, given that symptom relief is fundamental in high-quality palliative care and has a strong influence on patients’ quality of life.^27^ Therefore, incorporating role-play into training programs that address both communication and alleviating symptom is potentially useful, even when delivered online. The minimal clinically important difference remains unknown; thus, the small observed difference may not be clinically meaningful. However, comments such as “After this training, I was able to interact with patients who were worried about their future treatment, and acknowledge their pain by imagining their holistic suffering,” suggest that this program had at least some educational effect.

In addition, the significant reduction in perceived difficulty in communicating with the patient and family indicates that the 90-minute role-play session was effective, consistent with previous studies utilizing interactive workshops and distributing evaluation tools.^28^ Further research is warranted to assess the sustainability of such improvements.

The observed increase in confidence may be explained by the interactive nature of role-play, which enhances learners’ confidence in face-to-face settings;^29^ this benefit appears to be retained even in online formats. Supporting this, participants shared comments one month after the program. Some of the comments were “I was able to listen to the patient’s thoughts and feelings and have an empathetic attitude” and “I began to write down the intensity of the pain, the situation, and the evaluation after the use of painkillers in detail.” Thus, the program may have contributed not only to improving confidence but also to applying skills in clinical practice.

Participants reported being highly satisfied with the program. In particular, items such as “The content of the training was suited to my needs” and “The level of difficulty of the training was appropriate” received high ratings; therefore, the program content and difficulty appeared to be well-matched to the learning needs of nurses with two to four years of experience. Although the online format could have posed challenges such as unstable Internet connectivity,^30^ the participants found the learning environment smooth and largely free of technical difficulties. Furthermore, while role-play activities sometimes cause discomfort or embarrassment,^31^ participants gave high ratings to items such as “There was an appropriate level of tension” and “I was able to discuss with my peers and analyze what was good and what was lacking.” Thus, the program may have fostered a psychologically safe and engaging learning environment, enabling the participants to actively challenge themselves in a supportive setting.

This study has several limitations. First, 12 out of 43 participants dropped out, including 9 before baseline assessment. Common reasons included lack of communication, poor Internet connectivity, and technical issues—factors specific to online learning. To reduce dropout, future studies should implement strategies such as providing technical support and sending regular reminders.^32^ Second, given the single-group pre–post study design, causal relationships cannot be determined, and the results are not generalizable. Participation was voluntary, which may have introduced selection bias. Also, confounding factors such as additional study, training, and clinical experience in the month after the intervention could have influenced the results. Third, some of the instruments used, particularly those assessing confidence and the level of practice in evaluating researchers, have not been psychometrically validated. Moreover, the study did not evaluate whether reduced difficulty and improved confidence and skills led to better patient and family outcomes. Future research, ideally within a randomized controlled trial design, should incorporate validated measurement tools and patient- and family-centered outcomes.

## Conclusion

This study demonstrated that a fully online palliative care education program incorporating interactive role-play effectively reduced early-career nurses’ perceived difficulties in symptom alleviation and communication. As supported by observational ratings and qualitative feedback, this intervention also enhanced nurses’ confidence and application of learned skills in clinical practice. High levels of satisfaction further affirm that the program is feasible and acceptable. Therefore, brief, interactive online training may be a viable and scalable strategy to help early-career nurses develop essential palliative care competencies. Future research should employ randomized controlled trials and incorporate patient- and family-centered outcomes to assess the long-term impact and clinical relevance of this online program.

## Data Availability

All data relevant to the study are included in the article.

## Abbreviation used

ELNEC: End-of-Life Nursing Education Consortium
ELNEC-J: End-of-Life Nursing Education Consortium-Japan
CNS: certified nurse specialist
PCDS: Palliative care difficulties scale
NDCC: Nurses’ difficulty with cancer care
PCPS: Palliative care self-reported practices scale
OSCE: Objective Structured Clinical Examination
SD: Standard deviations
